# A Case Report of Deterioration in a Non-frail Octogenarian Burn Patient

**DOI:** 10.7759/cureus.71808

**Published:** 2024-10-18

**Authors:** Manya Bali, Denise Nadora, Olivia Wu, Kaila Polizzi, Eldo Frezza

**Affiliations:** 1 Medicine, College of Medicine, California Northstate University, Elk Grove, USA; 2 Surgery, College of Medicine, California Northstate University, Elk Grove, USA

**Keywords:** burn protocol, elderly burns, emergency department, frailty, geriatrics, octogenarian

## Abstract

While advancements in critical care and burn treatment have improved over the decades, elderly burn victims continue to face high mortality rates. Measurements of frailty among patients have become popular tools for predicting burn outcomes over chronological age. In this report, we provide a case of a non-frail octogenarian burn victim who deteriorated rapidly during treatment, suggesting that frailty alone is not sufficient in predicting outcomes in older burn patients.

An active 86-year-old male with hypertension presented to the emergency department with 35% total body surface area (TBSA) burns following a welding accident. He experienced second and third degree burns to his face, thorax, chest, back, and arms and had possible inhalation injury. Despite wound cleaning and fluid resuscitation, the patient’s vitals and pain worsened while waiting for transfer to the burn unit, requiring an oxygen mask and intravenous hydromorphone to be administered multiple times. In the emergency department (ED), the patient also experienced myoglobinuria, decreased urine output, and progressive confusion.

Frailty involves understanding how patient comorbidities and functional status influence the body’s ability to respond to stressors. Unlike their younger counterparts, octogenarian patients appear to be vulnerable to worse burn outcomes even when non-frail. Thus, physicians should consider injury severity and systemic responses to injury on admission in addition to an elderly patient’s pre-burn physiology to guide prognosis and treatment.

## Introduction

In the United States, nearly 400,000 people are treated for burns annually, and approximately 30,000 of those patients are hospitalized for burn-related injuries each year [1]. In recent decades, outcomes for burn victims have improved across all age groups due to advancements in critical care treatment, yet mortality among elderly patients remains high. From the age of 55 to 70 years, the mean lethal dose (LD50) decreases from 45% total body surface area (TBSA) burns to only 25% TBSA [2].

While many studies have established increased age as a predictor for burn mortality and decreased long-term function [3,4], there has been a recent interest in understanding how frailty scores may provide a more comprehensive assessment of elderly burn outcomes [5,6]. Frailty is a state of physiological vulnerability in which multiple body systems have reduced reserve or ability to function when faced with stressors [7]. A single standardized frailty scale has not yet been adopted, but the Canadian Study for Health and Aging Clinical Frailty Score (CFS) and the Modified Frailty Index-5 (mFI-5) are the commonly used assessments [6]. Both scoring systems utilize functional status/independence and comorbidities to stratify patients [8,9].

In this paper, we present a case of an 86-year-old patient who suffered a 35% TBSA burn. Despite his non-frail constitution and appropriate treatment by burn protocol, the patient’s condition declined rapidly, suggesting that factors beyond frailty should be considered by emergency providers.

## Case presentation

**Figure 1:**
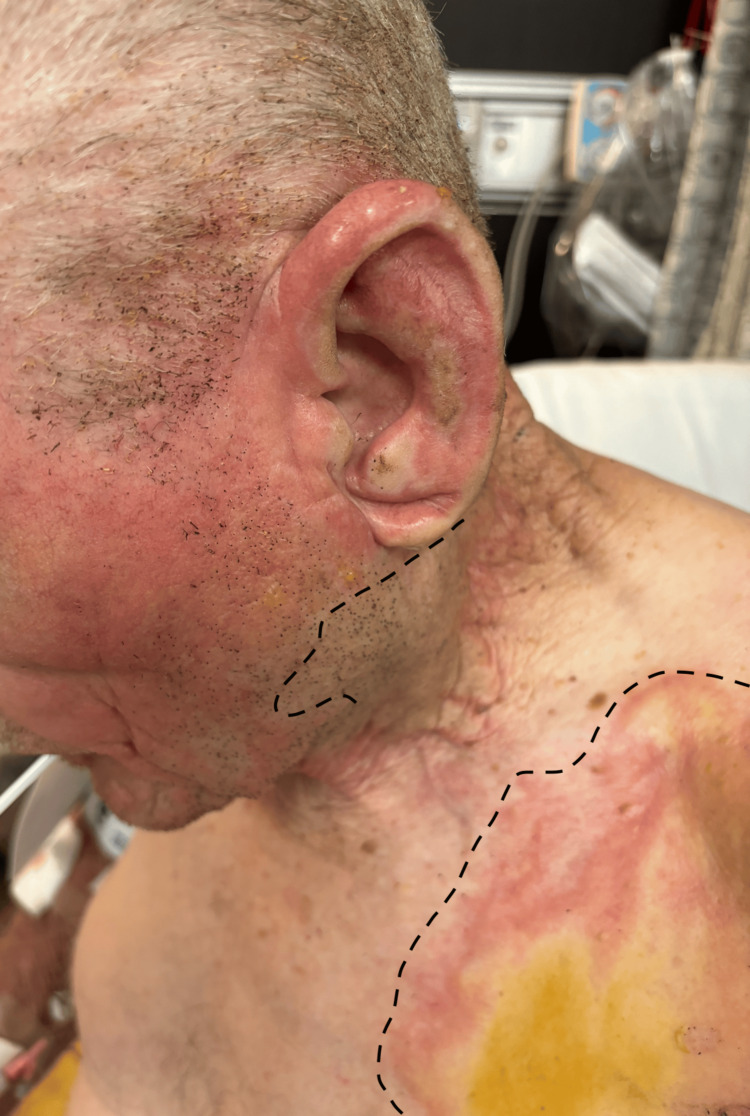
Burns of the left face and upper chest Burn boundaries are demarcated by the dotted lines.

An 86-year-old male presented to the emergency department (ED) due to a burn while welding in his yard. A spark ignited his shirt and caused burns on his face (shown in Figure 1), chest, upper back, and upper arms with hair burned at the level of the nose and possible CO_2_ inhalation. Over the next two hours, his blood pressure decreased from 140/100 to 120/80 to 110/70, while his heart rate continued to increase from 99 to 101 to 120 beats per minute. His oxygen saturation was 86% without supplementation. Medical history was unremarkable except for hypertension, which is controlled by lisinopril 10 mg. Vital signs are summarized in Table 1.

**Table 1:** Progression of vital signs

	First Reading - Upon Arrival	Second Reading - 30 minutes	Third Reading - 90 minutes	Reference Range
Blood pressure	140/100	120/80	110/70	120/80
Heart rate	99	101	120	60-100

**Figure 2:**
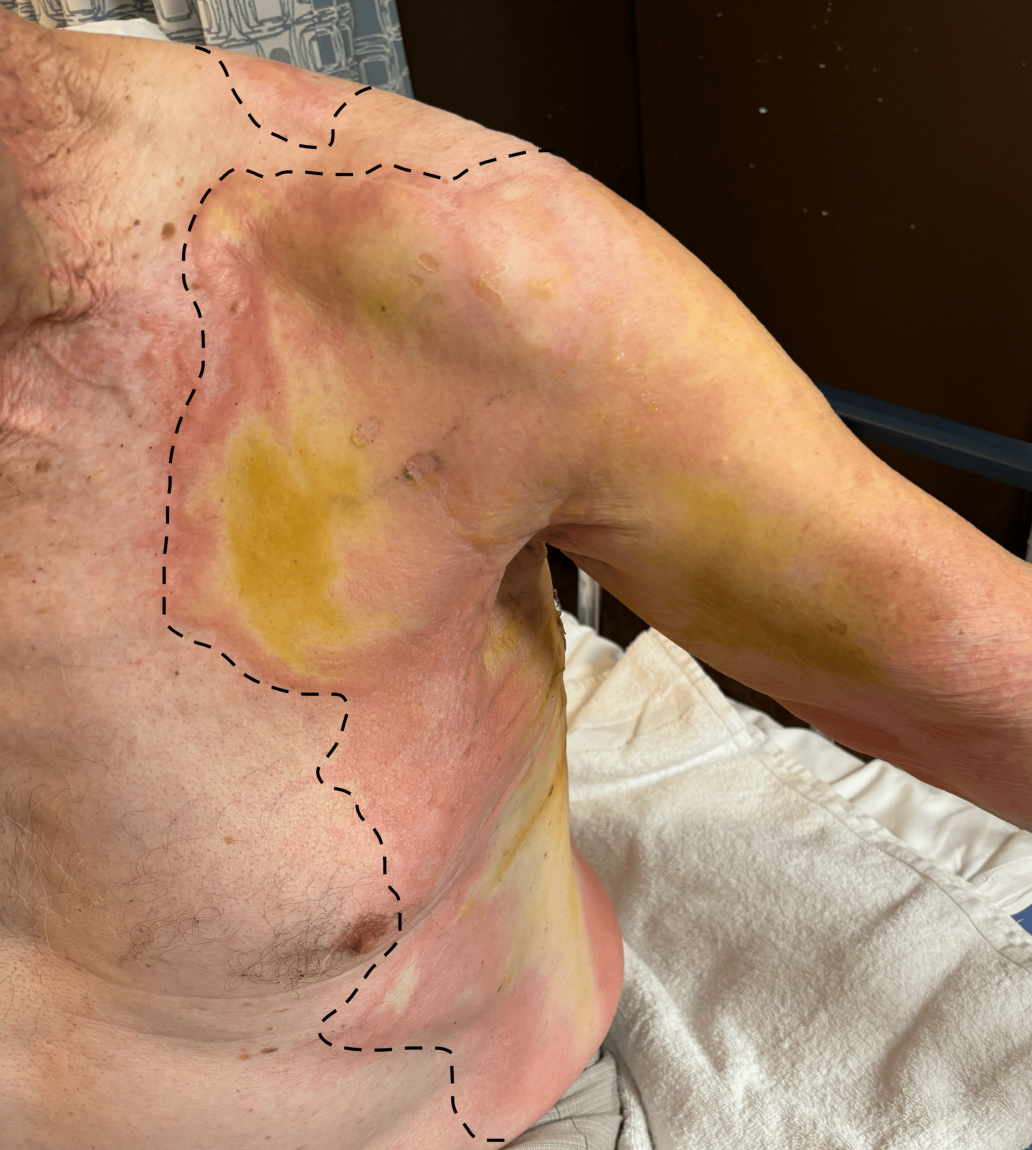
Burns of the chest and arm Burn boundaries are demarcated by the dotted lines.

**Figure 3:**
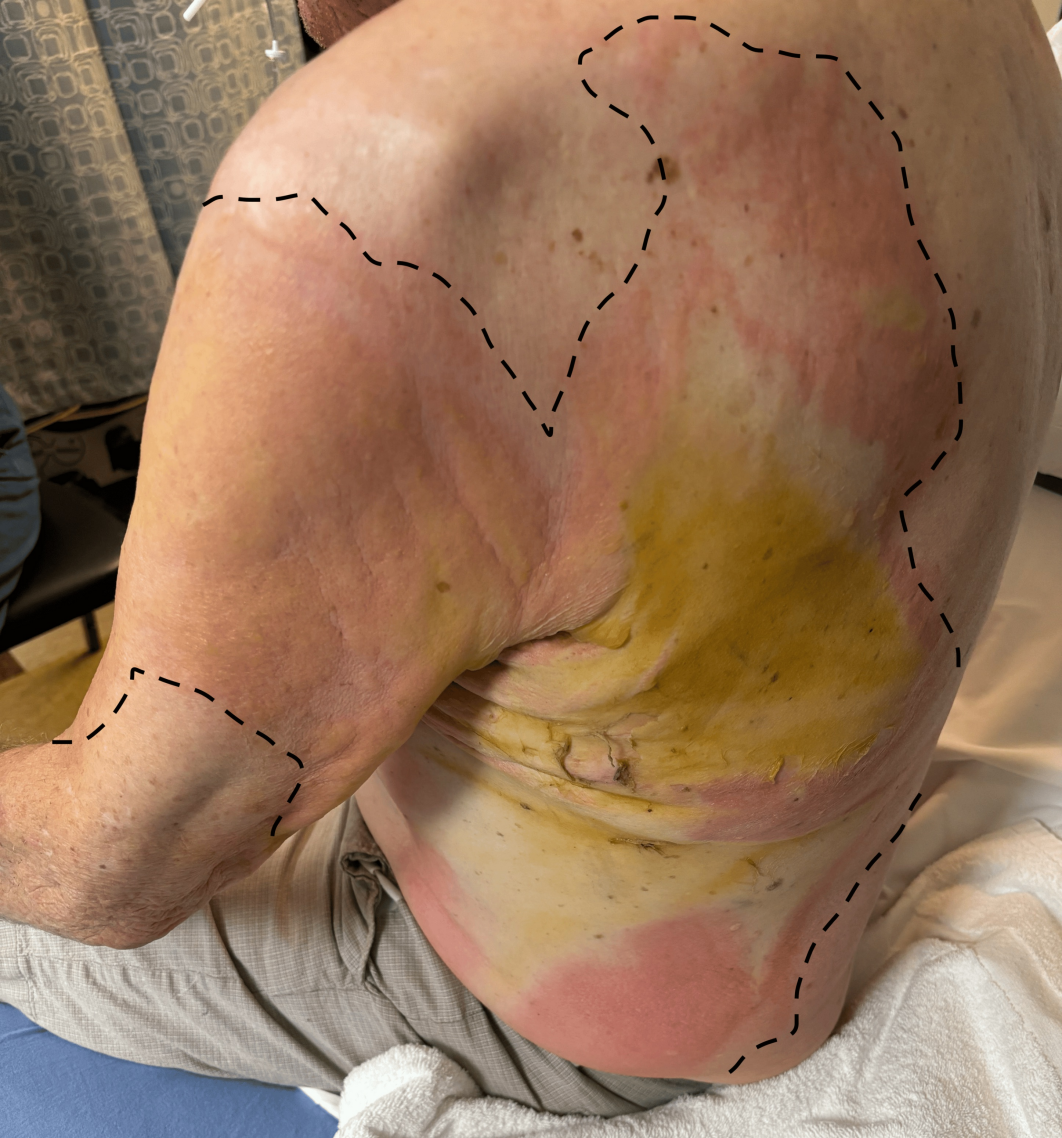
Burns of the back Burn boundaries are demarcated by the dotted lines.

Upon physical exam, he was found to have second and third degree burns covering 35% of his body with demarcated painful and painless areas on the chest and leathery burns on his back (Figures 2, 3). The patient was awake, alert, and ambulatory upon admission (GCS 15), but the patient became progressively confused with time and location within one hour of admission. Carboxyhemoglobin levels were at 9 percent. A chest x-ray showed no pulmonary complications despite the hair burn at the level of his nose. An electrocardiogram was performed, which showed a normal sinus rhythm. Additional laboratory findings upon arrival are summarized in Table 2.

**Table 2:** Laboratory findings upon arrival

	Value	Reference Range
White Blood Cell count (WBCs/µL)	19,000	4,500-11,000
Hemoglobin (g/dL)	14	13.8–17.2
Creatinine (mg/dL)	0.7	0.7-1.3
Urinalysis	Myoglobinuria	N/A

The patient was placed on a 2-liter nasal cannula, which increased his oxygen saturation to 90%. He underwent fluid resuscitation according to the Parkland formula for four hours prior to transfer to the burn unit. His wounds were cleaned with water and treated with silver sulfadiazine. Additionally, a Foley catheter was placed.

By the time of transfer, the patient's pain level had worsened, requiring intravenous hydromorphone to be administered three times. The color of his urine, initially yellow, changed to a darker, orange color. He also required 5-liter of O_2_ by mask, resulting in an oxygen saturation of 94%. A bronchoscopy was performed and was negative for inhalation injury. From the burn unit, the patient was then transferred to the intensive care unit (ICU) where he remained for three days. After an additional three days in the wards, he was discharged home.

## Discussion

Frailty can be considered a distinct geriatric syndrome that increases risk of adverse health conditions, comorbidities, physical and mental decline, fall-related accidents, and mortality [10]. The scoring guidelines of two commonly used assessments, the CFS and mFI-5, in burn studies are listed in Tables 3, 4, respectively [8,9].

**Table 3:** A summary of the Clinical Frailty Score (CFS)

Clinical Frailty Scale
1 Very Fit: active, energetic, and motivated with regular exercise
2 Well: no active disease and occasional activity
3 Managing Well: well-controlled medical problems and some activity beyond walking
4 Vulnerable: independent but symptoms limit activities
5 Mildly Frail: evident slowing and need help with high-order activities of daily living (ADLs)
6 Moderately Frail: needs help with all outside activities and in the house
7 Severely Frail: completely dependent for personal care
8 Very Severely Frail: completely dependent and approaching end of life
9 Terminally Ill: life expectancy of less than six months and not evidently frail

**Table 4:** Modified five-item Frailty Index (mFI-5)

Modified 5-Item Frailty Index
Diabetes mellitus
Congestive heart failure
Hypertension requiring medication
History of chronic obstructive pulmonary disease (COPD) or pneumonia
Non-independent functional status

The CFS is perhaps the most well-studied index of frailty in burn patients; multiple investigations have found that a CFS score of 5 or higher in the elderly is associated with greater mortality [11-13]. Interestingly, this conclusion is not found in all studies. Sepehripour et al. reported that CFS scores were correlated with treatment complications but not life expectancy in burn patients [14]. Using the mFI-5, one study of 574 patients found that frailty was not associated with mortality or complications but was correlated with longer hospital stay in burn victims aged over 65 years [15].

Our patient, an active octogenarian with well-controlled hypertension, would not be classified as frail under these guidelines, scoring a 3 on the CFS and a 1 on the mFI-5. Nonetheless, his condition declined considerably despite emergency treatment. For those over the age of 50, any burn greater than 10% TBSA is classified as severe and can cause significant systemic inflammation and intravascular fluid loss, leading to hypovolemia and hypoperfusion [16]. The presence of burned nostril hair in our patient suggests a degree of nasal inhalation injury despite a normal chest x-ray and bronchoscopy, and inhalation injury also classically increases mortality risk [17]. With his age, 35% TBSA, inhalation injury, and myoglobinuria, our patient clearly sustained a high level of injury that depleted his physiological reserve.

All elderly patients, regardless of frailty, will experience some level of age-related decreased pulmonary function, body mass, skin thickness, circulation, and immune response which can contribute to worse burn outcomes [18]. Considering the progression of our patient, providers should be cautioned against using frailty metrics alone as prognostic tools for burns. Adding additional factors to frailty indices beyond comorbidities and functional status appears to increase their accuracy in predicting burn outcomes. A report by Maxwell et al. developed a burn-specific frailty index that accurately predicted morbidity and mortality in elderly frail patients by including mental health and burn severity on admission into its scoring system in addition to patient comorbidities and functional status [19]. A separate analysis of over 1000 patients used the following factors to score frailty: age > 70 years, body mass index <18.5 kg/m^2^, hematocrit <35%, albumin <3.4 g/dL, and creatine >2.0 mg/dL. Patients with three or more risk factors had increased 90-day mortality and prolonged ICU stay [20]. Although these study-specific indices can be more difficult to calculate on admission, understanding the combined role of age, frailty, injury severity, and the systemic response of burn patients may more accurately guide prognosis and treatment management.

## Conclusions

Elderly patients have a decreased ability to respond to external stressors such as burns as compared to their younger counterparts. This contributes to higher complication and mortality rates among elderly burn patients. Although frailty indices consider patient comorbidities and functional status, they may not be sufficient in predicting outcomes, especially among octogenarians as shown in this case. Physicians should understand aspects of an elderly patient’s pre-burn physiology as well as their injury severity and systemic responses on admission to guide prognosis and treatment.
